# Lipid droplets in Zika neuroinfection: Potential targets for intervention?

**DOI:** 10.1590/0074-02760230044

**Published:** 2023-10-09

**Authors:** Suelen Silva Gomes Dias, Tamires Cunha-Fernandes, Vinicius Cardoso Soares, Cecília JG de Almeida, Patricia T Bozza

**Affiliations:** 1 Fundação Oswaldo Cruz-Fiocruz, Instituto Oswaldo Cruz, Laboratório de Imunofarmacologia, Rio de Janeiro, RJ, Brasil; 2 Fundação Oswaldo Cruz-Fiocruz, Centro de Pesquisa, Inovação e Vigilância em COVID-19 e Emergências Sanitárias, Rio de Janeiro, RJ, Brasil; 3 Universidade Federal do Rio de Janeiro, Programa de Imunologia e Inflamação, Rio de Janeiro, RJ, Brasil

**Keywords:** lipid metabolism, lipid droplets, immunometabolism, inflammation, Zika virus, neuroinfection

## Abstract

Lipid droplets (LD) are evolutionarily conserved lipid-enriched organelles with a diverse array of cell- and stimulus-regulated proteins. Accumulating evidence demonstrates that intracellular pathogens exploit LD as energy sources, replication sites, and part of the mechanisms of immune evasion. Nevertheless, LD can also favor the host as part of the immune and inflammatory response to pathogens. The functions of LD in the central nervous system have gained great interest due to their presence in various cell types in the brain and for their suggested involvement in neurodevelopment and neurodegenerative diseases. Only recently have the roles of LD in neuroinfections begun to be explored. Recent findings reveal that lipid remodelling and increased LD biogenesis play important roles for Zika virus (ZIKV) replication and pathogenesis in neural cells. Moreover, blocking LD formation by targeting DGAT-1 *in vivo* inhibited virus replication and inflammation in the brain. Therefore, targeting lipid metabolism and LD biogenesis may represent potential strategies for anti-ZIKV treatment development. Here, we review the progress in understanding LD functions in the central nervous system in the context of the host response to Zika infection.

Lipid droplets (LD) are complex and dynamic organelles centrally involved in energy and lipid homeostasis, membrane biosynthesis, cell signalling and the production of inflammatory mediators and antimicrobial compounds.^1^ ^,^ ^2^ Structurally, LD are endoplasmic reticulum (ER)-derived organelles composed of a core of neutral lipids (triacylglycerol, diacylglycerol and cholesterol ester) surrounded by a monolayer of phospholipids associated with a diverse composition of proteins.^3^

It is now well established that LD exert major functions in inflammatory and infectious diseases. The participation of LD in infectious disease pathogenesis has been reported for all classes of pathogens, such as viruses, bacteria, fungi and protozoa, suggesting that LD participate in the innate and adaptive host immune response to infection.^4^ Host LD may also be exploited as part of the adaptation of pathogens to escape the immune system and as an energy source for intracellular pathogens.^4^

Contemporary evidence supports important functions for lipid metabolism and LD in the central nervous system (CNS).^5^ ^,^ ^6^ ^,^ ^7^ ^,^ ^8^ Indeed, different cells of the brain exhibit LD in the context of development, obesity, and neurodegenerative pathologies.^5^ ^,^ ^9^ ^,^ ^10^ Although LD play major roles in the pathogenesis of leprosy neuropathy in the peripheral nervous system (reviewed in^11^), only recently have studies started to unveil the functions of CNS LD in the context of infection. Here, we review the emerging evidence of the functions of LD in infections of the CNS, focusing on LD functions in Zika neuroinfection.

*Lipids in the context of neurological disorders* - The CNS has a large amount and diversity of lipids, and the metabolism of these biomolecules plays an essential role in initial development, myelin sheath formation, and signalling.^12^ Therefore, changes in the lipid metabolism and LD biogenesis of CNS cells can lead to cell dysfunction and are associated with inflammation, aging, obesity, and the development of neurodegenerative pathologies.^5^ ^,^ ^9^ ^,^ ^13^

The development of neurodegenerative diseases has been associated with the gradual loss of neurons associated with a reduction in antioxidant capacity and an increase in oxidative stress. Moreover, mitochondrial dysfunction may result in increased production of reactive oxygen species (ROS), contributing to the increase in LD as well as lipid peroxidation in Drosophila and mice.^10^

Alterations in lipid metabolism and accumulation of LD have been associated with neurodegenerative diseases, causing the early onset of defects in neurogenesis and affecting the regeneration and plasticity of neural progenitor cells, as observed even before the deposition of amyloid plaques in Alzheimer’s disease.^14^ Likewise, Parkinson’s disease is also associated with alterations in lipid metabolism pathways. Parkinson’s disease is often associated with the formation of cytoplasmic inclusions caused by the aggregation of α-synuclein. This process depends on interactions of α-synuclein with phospholipids and fatty acids, provoking an excessive membrane interaction, which stimulates aggregation.^15^ Moreover, alpha-synuclein interacts with LD and changes TAG metabolism. The pathogenesis of the disease may occur due to dysfunction or overexpression of this protein, and this association with LD could contribute to the development of the disease.^16^

In different cells, organs and systems LD play major functions in inflammation and host-response to infection.^4^ Accumulating evidence indicate that LD are also involved in inflammation in the CNS. Indeed, LPS triggers increased LD formation in vitro and *in vivo* microglia.^17^ ^,^ ^18^ LPS-induced microglia LD co-localised with cytosolic phospholipase A_2_, a key enzyme for arachidonic acid release, suggesting roles for LD in eicosanoid synthesis in activated microglia, as it has been demonstrated for different cells of the immune system.^19^ Moreover, microglia enriched in LD were demonstrated to produce high levels of reactive oxygen species and to secrete increased amounts of proinflammatory cytokines.^18^ However, whether LD in glia are hubs for neuroinflammation remains to be better defined.

*Zika virus (ZIKV) infection and pathogenesis in the CNS* - The arbovirus ZIKV belongs to the family *Flaviviridae* and genus Flavivirus. The ZIKV structure comprises a nucleocapsid and an icosahedral symmetric structure with a diameter of approximately 50 nm.^20^ The ZIKV genome consists of a single-stranded ribonucleic acid (RNA) with positive polarity (+RNA) and an approximate size of 11 kb. The genome encodes a polyprotein, which is cleaved to generate three structural proteins - the capsid protein (C), the premembrane/membrane protein (prM/M), and the envelope protein (E) - and seven nonstructural proteins (NS) - NS1, NS2A, NS2B, NS3, NS4A, NS4B, and NS5. These proteins are involved in replication and control host cell processes to allow virus maintenance.^21^

Although the symptoms of ZIKV infection are generally self-limited, with fever, rash, headache, and conjunctivitis, it is of great concern in the case of infected pregnant women.^22^ In the late 2000s, ZIKV re-emerged dramatically, marked by the rapid epidemic wave that devastated America, which characterised an international public health emergency declared by the World Health Organization (WHO).^23^

The association of ZIKV infection with the development of alterations has already demonstrated neurological complications in neonates, such as microcephaly and other severe congenital CNS malformations.^24^ In addition, ZIKV infection is associated with the development of Guillain-Barré syndrome (GBS) in adults. This neurological complication may lead to peripheral nerve impairment and respiratory failure.^25^

ZIKV infection can trigger congenital changes with a spectrum of neurological dysfunctions characterised by congenital ZIKV syndrome in foetuses and babies infected during pregnancy. Such neurological complications include severe microcephaly, deep skull depressions, parenchymal or cerebellar calcifications, apparent tissue damage to the brain, scarring or pigment changes in the back of the eyes, atrophy, and joints with limited range of motion after birth. These effects of ZIKV on foetuses are usually more frequent and severe when maternal infection occurs in the first and second trimesters of pregnancy, resulting in miscarriage and an increased likelihood of congenital malformation, foetal death, and neonatal and postneonatal deaths.^26^ ^,^ ^27^

During ZIKV infection, neural changes are associated with the death of neural progenitor cells and mature neurons, albeit to a lesser extent.^28^ Therefore, understanding the pathogenesis of ZIKV is extremely important to prevent the irreversible and severe damage observed, particularly in foetuses during pregnancy, compromising neurological development. Several response mechanisms are activated by host cells during ZIKV infection, such as the production of inflammatory mediators, activation of signalling pathways, and autophagy, which can lead to cell depletion and compromise cell viability due to the metabolic reprogramming observed during the infection.^29^

ZIKV infection causes changes that contribute to damage in neurological development. Brains of ZIKV-infected mice show a significant amount of infectious particles compared to other organs, corroborating the tropism of ZIKV for neural cells.^30^ Furthermore, ZIKV infection significantly reduces the body and brain size of mouse foetuses. Surviving mice exhibit cortical malformations associated with a delay in the cell cycle, a lower number of cells, cell death, and diminishment of the thickness of the cortical layer. Thus, ZIKV infection affects neuronal development in mice, similar to the outcome of microcephaly in humans.^30^ ^,^ ^31^

ZIKV infection also induces microglial activation, which contributes to the immune response to the pathogenesis of ZIKV infection in the developing brain.^32^ Despite all the severe outcomes of ZIKV infection, there is still no specific treatment for the disease, and there are no vaccines available against this virus. Given this, it is necessary to understand more about the mechanisms involved in the infection and pathogenesis to envisage prevention and control of the disease.

*Functions of LD in ZIKV neuroinfection* - There is a global concern about developing antiviral therapies to control these mechanisms in this scenario. Although ZIKV has been known for decades, antiviral therapies against it are still lacking and facing enormous challenges, especially regarding how to block transmission from pregnant women to their foetuses.^33^ Currently, therapies are focused on the search for direct acting antivirals that interfere with viral host components that hamper virus assembly and release.^34^

Lipids are essential constituents of the cell membrane, providing structure and energy for living organisms. Lipidomic and transcriptomic analyses reveal that ZIKV infection reprograms lipid metabolism and alters the relative amounts of different lipid classes, such as sphingolipids, glycerophospholipids, plasmalogens, lysophospholipids, and (di)carboxylic acids. This class of biomolecules also plays other roles during the inflammatory process, such as acting as signalling molecules that modulate the inflammatory response.^35^ Markedly, reprogramming of lipid metabolism is central for virus entry, replication, assembly, and release.^33^ ^,^ ^36^ ^,^ ^37^

Flavivirus infection induces extensive membrane remodelling, creating niches that protect the viral genome from degradation and allow viral assembly. There is also stimulation of the biogenesis of LD, which are often located close to the membrane vesicles containing the viral replication complexes, contributing to the replication and assembly of ZIKV and other flaviviruses.^38^ ^,^ ^39^ ^,^ ^40^ Similar to dengue virus (DENV) and hepatitis C virus (HCV),^38^ ^,^ ^39^ ^,^ ^40^ ZIKV capsid (C) protein associates with LD^41^ ^,^ ^42^ giving support to LD role as ZIKV replication platform and generation of virus particles. These processes require de novo lipid synthesis and lipid mobilisation. In fact, viral infection relies on rewiring host metabolism, especially lipid metabolism.^4^ ^,^ ^43^

LD are the main lipid storage site found in almost all prokaryotic and eukaryotic cells and present an association with several proteins that confer different functions to this organelle.^3^ ^,^ ^44^ LD present a monolayer of phospholipids associated with several structural proteins (perilipin 1-5) and exhibit a core rich in neutral lipids. They act in fatty acid trafficking and lipid signalling. In addition, LD harbour the machinery to produce inflammatory lipid mediators, such as prostanoids.^45^ ^,^ ^46^ During infections, LD can be viewed as a double-edged sword since they support viral replication and contribute to the production of metabolites of the host defence.^47^ ^,^ ^48^

The mechanisms of formation and functions of LD in Zika infections of neuronal cells are starting to be unveiled. We have recently shown that ZIKV usurps the host metabolism of human neuronal cells SH-SY5Y (neuroblastoma cell line) and in NSCs derived from iPSCs, stimulating the expression of lipogenic genes, such as DGAT-1, FASN, PPAR-γ, SREBP-1, and PLIN-2, while decreasing the expression of lipolytic enzymes, such as HSL and ATGL.^40^ This lipogenic profile agrees with the increase in LD. Notably, the inhibition of acyl-CoA:diacylglycerol acyltransferase-1 (DGAT-1) with A922500 impairs LD production and viral replication in these cells.^40^

Sterol regulatory element-binding protein (SREBP) is an essential lipid metabolism regulator. It regulates cholesterol and lipid homeostasis and has been associated with LD biogenesis in several viral infections, including HCV, DENV, and ZIKV.^33^ ^,^ ^49^ ^,^ ^50^ SREBPs are ER-residing transcription factors that control the expression of key lipogenic enzymes, thereby integrating multiple cellular signals to control lipogenesis and subsequent LD biogenesis.^51^ They are synthesised as precursor proteins and need processing to be activated. In the ER, SREBPs interact with SREBP-cleavage activating protein (SCAP), which is a sensor of the cholesterol level in the ER. Binding to cholesterol promotes SCAP interaction with insulin-induced genes 1 and 2 (INSIGs). When cholesterol levels are low, SCAP becomes free to interact with COPII and translocate SREBPs to the Golgi, where site 1 protease (SKI-1/S1P) and site 2 protease (S2P) cleave it. Then, the soluble N-terminus of SREBP goes to the nucleus and regulates the expression of genes involved in lipid synthesis, such as cholesterol and fatty acids.^52^ ^,^ ^53^

Flaviviruses interact with other cholesterol biosynthetic machinery components, such as SCAP. In DENV infection, STING, a part of the interferon-signalling pathway, is cleaved by the viral NS2B3 protease complex. SCAP impairs the ability of NS2B3 to cleave STING, counteracting the viral strategy to escape host immunity. Knockdown of SCAP facilitates, whereas SCAP overexpression blocks, DENV infection.^54^ Another study showed that PF-429242 (an inhibitor of SKI-S1P) inhibits DENV infection, an effect that is reversed by loading cells with oleic acid, an inducer of LD biogenesis.^50^ Furthermore, the SREBP inhibitor AM580, an agonist of retinoic acid, presents antiviral activity against ZIKV infection, reducing LD biogenesis and the production of infectious particles.^55^ In this context, inhibiting SREBP activation is a good candidate for reducing lipid metabolism and diminishing ZIKV infection.

Fatty acid metabolism is also important for ZIKV infection. According to the alteration of FASN expression observed during ZIKV infection, INH-12, a hydroxysteroid (17β) dehydrogenase (HSD17B) inhibitor, promotes a reduction in LD biosynthesis, diminishing the release of infectious particles of ZIKV, DENV and HCV. HSD17B is a key enzyme of the steroid metabolism pathway and the second step for very long-chain fatty acid synthesis (VLCFA). HSD17B knockdown also diminishes VLCFAs and the production of HCV infectious particles, whereas the addition of oleic acid restores LD formation and the production of infective particles.^56^ DENV also alters fatty acid metabolism. One such mechanism is recruiting FASN by viral NS3. Interestingly, de novo lipid synthesis localises to viral replication complexes.^57^ HCV infection, or the expression of protein C and NSB4, also stimulates FASN expression and activity, resulting in increased levels of triacylglyceride, which allows HCV replication^58^ and increases VLCFAs.^59^ The synthesis of very long fatty acids from the elongation of palmitate is a process that takes place in the ER.

Beyond serving as platforms for viral replication and assembly, LD are an essential source of lipids, which can be mobilised by lipophagy, which is associated with flavivirus infection.^57^ ZIKV promotes deubiquitination of the LD-residing ancient ubiquitous protein 1 (AUP1) and its autophagosome translocation. This event stimulates the acyltransferase activity of AUP1, upregulating lipophagy and promoting the generation of infectious viruses. AUP1 knockdown eliminates the production of infectious DENV and ZIKV virions.^60^ Alternatively, ZIKV may exploit LD to evade the immune response.^61^ The mechanism of the induction of LD biogenesis during ZIKV is still not precise and deserves further study.

ZIKV infection activates the mechanistic (mammalian) target of rapamycin (mTOR) pathway, a master regulator of autophagy, impacting neurogenesis. The NS4A and NS4B proteins are responsible for inhibiting the protein kinase B (PKB/AKT)-mTOR complex, diminishing the viability of human foetal neural stem cells and increasing ZIKV pathogenesis.^62^ ^,^ ^63^ ZIKV infection also activates mTOR complex I (mTORC1) and mTORC2 in neuronal and glial cells, and mTOR inhibition reduces ZIKV protein expression and the production of infectious particles.^64^ Interestingly, the timing of mTOR pathway activation determines the outcome of viral infection. Early activation of mTOR reduces the production of infectious particles and improves cell survival. However, late mTOR activation leads to cell death and the release of infectious particles.^64^ Lipophagy, in turn, is vital for providing energy for viral replication and highjacking the immune response.^60^ The multiple facets of autophagy during ZIKV are still poorly understood and worthy of better elucidation.

An important outcome of ZIKV infection is defective neuronal cell development and cell death, which may be associated with neurological complications.^62^ To evaluate the potential of LD inhibition to treat ZIKV infection *in vivo*, we infected neonatal mice (P2) with daily intraperitoneal injections of the DGAT-1 inhibitor A922500 for seven days. This treatment reduced viremia in different tissues, including the brain, hampered weight loss and rescued mice from death.

Finally, LD also contribute to the inflammatory response. ZIKV infection in astrocytes stimulates the production of LD and the release of IFN type I and III. Loading astrocytes with oleic acid before ZIKV infection increases the number of LD and further enhances the infection-stimulated release of IFN-β, IFN-γ and viperin, indicating that LD are a crucial cellular organelle in the antiviral innate immune response.^61^ ^,^ ^65^ The role of LD in the innate immune response agrees with our results using the *in vivo* ZIKV infection model, which shows that the DGAT-1 inhibitor A922500 decreases cytokine/chemokine production in addition to impairing LD production in human neuronal cells. Moreover, LD inhibition by targeting DGAT-1 *in vivo* also diminished the inflammatory response, as evidenced by the decrease in TNF-α, IL-1β, and MCP-1 in the brains of ZIKV infected treated animals compared to infected nontreated animals.^40^

Collectively, ZIKV modulates host lipid-related pathways leading to LD accumulation in different neuronal cells. Moreover, LD plays a critical role during the ZIKV cycle, serving as platforms for virus replication and sources of lipids for viral particle formation. Accordingly, strategies to inhibit LD formation significantly reduces virus replication in neuronal cells. As proof of concept, LD inhibition by DGAT-1 inhibition reduced the ZIKV particle load and inflammation in the brain and significantly protected against ZIKV-induced mortality in mice. Therefore, the pharmacological targeting of LD formation is a potential strategy for antiviral development in Zika infection (Figure).

**Figure:**
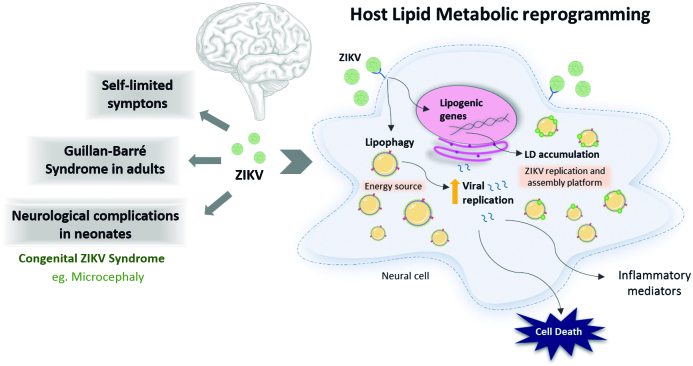
Neuropathogenesis and alterations in the host lipid metabolism during Zika virus (ZIKV) infection. Infection by ZIKV causes self-limited symptoms, such as fever, rash, headache, and conjunctivitis. Moreover, the infection leads to several neurological disorders in adults and neonates, such as Guillan-Baré syndrome and microcephaly, respectively. Looking at molecular levels, ZIKV infection induces lipid metabolism reprogramming in host cells as observed by increased lipogenic genes. Furthermore, ZIKV infection promotes lipid droplet accumulation that can be used as a replication and assembly platform. These phenomena together with lipophagy favour viral replication, promoting an increase of inflammatory mediators that can contributes to the neural cell’s death.

*Concluding remarks* - Emerging evidence places LD as key organelles in ZIKV pathogenesis in the CNS (Figure). However, critical questions remain about the formation and multiple functions that LD plays in neuroinfections. Further investigations should help us to decipher the full range of LD functions in the host protective immune response as well as to better understand pathogen-specific mechanisms evolved to take advantage of LD for their survival and the persistence of neuroinfections. In addition, LD are emerging as attractive target candidates for therapeutic intervention in infectious diseases that progress with increased LD accumulation. Future studies will need to include the development of selective LD inhibitors. Moreover, the safety characterisation of LD inhibition is needed, as lipid accumulation within LD may act as a protective mechanism in lipid homeostasis against cellular lipotoxicity.
